# An Overview of Machine Learning and 5G for People with Disabilities

**DOI:** 10.3390/s21227572

**Published:** 2021-11-14

**Authors:** Mari Carmen Domingo

**Affiliations:** Department of Network Engineering, BarcelonaTech (UPC) University, 08860 Castelldefels, Spain; cdomingo@entel.upc.edu

**Keywords:** machine learning, 5G, people with disabilities, wireless communication, network slicing architecture, applications, research challenges

## Abstract

Currently, over a billion people, including children (or about 15% of the world’s population), are estimated to be living with disability, and this figure is going to increase to beyond two billion by 2050. People with disabilities generally experience poorer levels of health, fewer achievements in education, fewer economic opportunities, and higher rates of poverty. Artificial intelligence and 5G can make major contributions towards the assistance of people with disabilities, so they can achieve a good quality of life. In this paper, an overview of machine learning and 5G for people with disabilities is provided. For this purpose, the proposed 5G network slicing architecture for disabled people is introduced. Different application scenarios and their main benefits are considered to illustrate the interaction of machine learning and 5G. Critical challenges have been identified and addressed.

## 1. Introduction

According to the World Health Organization 2011 report, over a billion people, including children (or about 15% of the world’s population), are estimated to be living with disability [1], and this figure is going to increase to beyond two billion by 2050 as the global population progressively ages and prevalence of non-communicable diseases rises [2].

People with disabilities generally experience poorer levels of health, fewer achievements in education, fewer economic opportunities, and higher rates of poverty than people without a disability [1]. Assistive technology can be crucial to improve the quality of life of people with disabilities, facilitate their equal participation in society, and make them more independent. Examples of assistive devices and technologies include wheelchairs, artificial limbs, hearing aids, visual aids, and specialized computer software and hardware that increase mobility, hearing, vision, or communication capacities. Assistive technology offers people with disabilities opportunities for work, education, leisure, social engagement, access to public services, and information. 

The Sustainable Development Goals (SDGs) are a collection of 17 global goals designed to address the global challenges we face, including those related to poverty, inequality, climate change, environmental degradation, peace, and justice [3]. The SDGs were adopted by all United Nations Member States in 2015, and they are intended to be achieved by the year 2030. Assistive technology for people with disabilities is essential for the equitable achievement of each of the 17 SDGs [4], such as universal health coverage, inclusive and equitable quality education, sustainable economic growth, decent work opportunities, reduction in inequalities paying attention to the needs of disadvantaged and marginalized populations, and creation of inclusive, safe, resilient, and sustainable cities.

**Figure 1:**
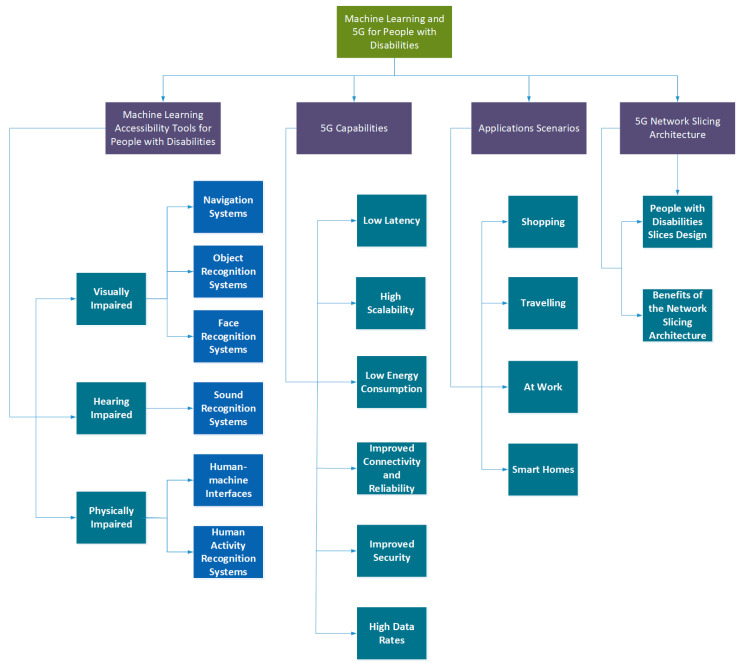
Proposed taxonomy of machine learning and 5G for people with disabilities.

Incentivizing research and development of assistive technology can help further accelerate the availability and dissemination of promising technologies to benefit people with disabilities [5]. Digital technologies, such as artificial intelligence and modern communication technologies (such as 5G), are disrupting nearly every sector of the economy [6]. These technologies can make major contributions towards all SDGs [7] and the assistance of people with disabilities so that they can achieve a good quality of life. Therefore, the purpose of this paper is to analyze how people with visual, hearing, and physical impairments can interact with and benefit from machine learning and 5G. To the best of our knowledge, this is the first paper that discusses machine learning, together with 5G, for people with disabilities. Figure 1 illustrates the taxonomy of machine learning and 5G for people with disabilities. The proposed taxonomy is based on the following components: (a) machine learning accessibility tools for people with disabilities, (b) 5G capabilities, (c) application scenarios, and (d) 5G network slicing architecture. In the next sections, we present the different components of this taxonomy in greater detail.

The paper is structured as follows. In Section 2, we discuss machine learning for people with disabilities. In Section 3, we introduce 5G for people with disabilities. In Section 4, the application scenarios and main benefits of machine learning and 5G for people with disabilities are described. In Section 5, we explain the 5G service-based architecture. In Section 6, we discuss the proposed 5G network slicing architecture for disabled people, from a technical perspective, and its main benefits. In Section 7, the main research challenges for machine learning and 5G for people with disabilities are outlined. Finally, the paper is concluded in Section 8.

## 2. Machine Learning for People with Disabilities

**Figure 2:**
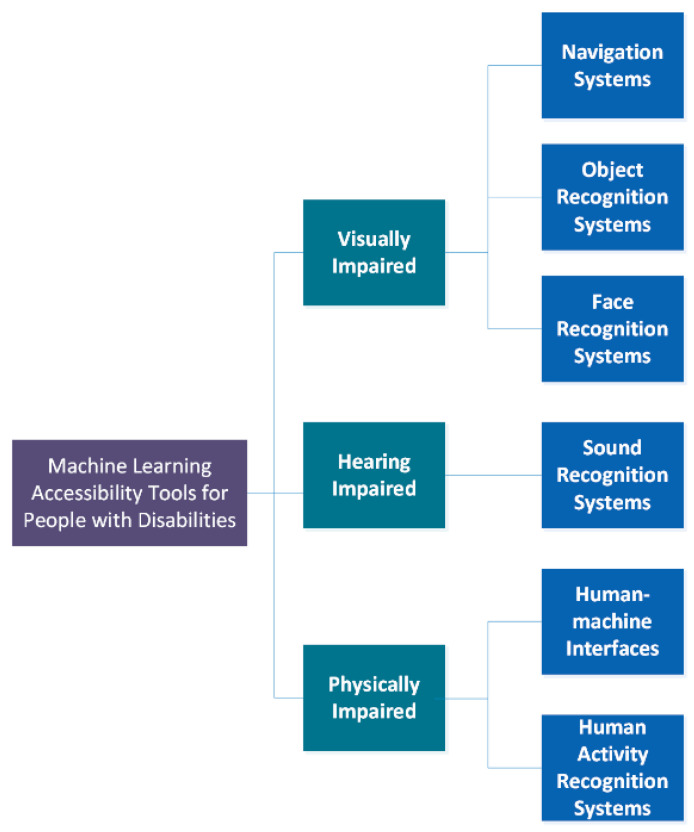
Proposed taxonomy of machine learning accessibility tools.

Artificial Intelligence (AI) refers to machines working in an intelligent way. Machine learning is a type of AI that provides machines with the ability to learn without being explicitly programmed [8]. Machine learning technology leverages advances [9] in computer vision, speech recognition, and auditory scene analysis to sense, interpret, and offer new ways to help people with disabilities. The taxonomy of the machine learning accessibility tools for people with disabilities (visually, hearing, and physically impaired) is illustrated in Figure 2. Next, we describe, in greater detail, the components of this taxonomy.

### 2.1. Visually Impaired

Artificial intelligence, with special emphasis on computer vision algorithms, has enabled the development of new systems to increase the mobility and personal autonomy of the visually impaired. The components designed for the visually impaired are: (1) *navigation systems*, (2) *object recognition systems*, and (3) *face recognition systems*. Next, these components are introduced.

#### 2.1.1. Navigation Systems

A *walking guide* [10] enhances the mobility of the visually impaired. It consists of two parts: an *obstacle and a pothole detection system*. The presence of obstacles can be detected in the front, left, and right directions using ultrasonic sensors. Audio messages are transmitted in the presence of obstacles. Images captured by a camera are sent to convolutional neural networks (CNNs) to detect the presence of potholes and alert users with audio signals. 

A smartphone-based solution [11] helps the visually impaired *navigate along predefined paths in indoor and outdoor scenarios*. It does so by loading a previously recorded virtual path and providing automatic guidance along the route, through haptic, speech, and sound feedback. Augmented reality technology is used to eliminate the need for any physical support, whereas CNNs are trained to recognize objects or buildings and make it possible to access the digital contents associated with them. 

The *location of a visually impaired* is obtained with enough precision *in indoor navigation* [12]. The indoor environment is divided into microcells. Two-dimensional local coordinates are assigned to the vertices of each microcell. Data from inertial sensors (accelerometer, gyroscope, and magnetometer) of a smartphone is used to train a multilayer perceptron (MLP). The neural network returns the 2D local coordinates of the microcell, related to the position of the smartphone and its owner, with an accuracy of more than 94% and a positioning error of 0.65 m.

Furthermore, an *artificial intelligence edge computing-based assistive system* helps the visually impaired to safely *use zebra crossings* [13]. When a visually impaired pedestrian reaches a zebra crossing next to a traffic signal, an intelligent walking cane receives the current pedestrian traffic signal status and transmits it to a waist-mounted device. This device guides the visually impaired with audio messages. In addition, a video of the zebra crossing is recorded via the front camera (module) of the blind person’s smart sunglasses, and the video is transmitted in real-time to the proposed mounted device. Based on the video images, it is possible to detect zebra crossings and determine if the pedestrian is deviating from the crossing when he/she is passing through. If this is the case, warnings are transmitted to the earphones of the visually impaired so that he/she corrects his/her route to avoid an accident. The Inception V2 based convolutional neural network is used for real-time zebra crossing recognition. 

A mobile vision-based *staircase detection system in indoor environments* can also be added [14]. The database of staircases candidates (upstairs, downstairs, and negatives (i.e., corridors)) is collected using an RGB-D camera. The Support Vector Machine (SVM) multi-classifier is used for training and testing during staircase recognition. 

Furthermore, an *assistance system to support the navigation of the visually impaired in low structured environments* has also been proposed [15]. A safe walking direction is computed based on the images from a 3D stereo camera mounted at the front of a backpack. Three different convolutional neural network (CNN) architectures (U-Net, PSPNet, and LinkNet) that include several backbones (DenseNet, EfficientNet, Inception, Mobilenet v1 and v2, ResNet, and VGG16) are used to segment the traversable path. Vibrotactile feedback is sent to the visually impaired once the local collision free trajectory is computed.

In addition, a *navigation system* that helps the visually impaired *run* is also developed [16]. The 3D camera captures information from its environment; an Artificial Neural Network (ANN) analyzes the images to detect the path; finally, vibration motors generate the navigation feedback. 

Also, a computer vision-based system [17] *recognizes bus information* based on images captured by a camera at a bus stop. The arrival of a bus is detected using Histogram of Oriented Gradients (HOG)-based feature extraction and a Cascaded SVM classifier; the information of the bus route number is extracted from the top part of the bus facade using a scene text extraction algorithm. Bus detection, as well as route number recognition, is transmitted to the visually impaired via audio.

#### 2.1.2. Object Recognition Systems

A vision-based system to *differentiate objects with a similar tactile sensation* is developed [18]. It consists of three modules:

- *An input module*: the visually impaired specifies to which class an object belongs; touch is used to estimate the object’s size and shape, and/or sound is used to obtain a feedback on the object content.
- *A visual recognition module*: it analyzes images from a video stream, as well as the prior information provided by the visually impaired, and tries to identify particular visual features from the object. For this task, different image descriptors (descriptions of the visual features of the contents in images or videos) have been compared; bag-of-features (BoF) provides a higher recognition performance than other image descriptors.
- *An output module*: it provides feedback to the user.

Deep learning techniques for object detection have improved significantly in recent years [19,20]. The *wearable smart glasses recognize drug pills* using deep learning [21], so that safety in medication-use for the visually impaired with chronic diseases is improved. The proposed system consists of a pair of wearable smart glasses, an artificial intelligence (AI)-based intelligent drug pill recognition box, a mobile device app, and a cloud-based information management platform. The Quick Response (QR) code on a drug package is scanned using the mobile device app to obtain the associated medication information, which is uploaded in the cloud-based management platform. The proposed AI-based intelligent drug pill recognition box consists of an AI-based embedded edge computing module and a power amplifier with a micro speaker. This box receives the medication information and reminds the patient to take his/her medicine, when the time comes, using voice messages. When the patient picks up the selected drug pills, the camera embedded on the proposed smart glasses takes and sends an image to the recognition box to analyze if this is the right medicine. This box also transmits the medication-use records to the cloud-based management platform, so the patient’s family members or caregivers are able to check the medication status using a mobile app. Two deep learning modules are used for object detection: (1) Single Shot Detector (SSD) as an object position detection module and (2) ResNet as an image classification module.

In addition, a *deep learning-based approach able to detect and recognize bills and coins*, from images captured by smartphone cameras in complicated backgrounds, is introduced [22]. The suggested three-stage detection technology for new banknotes and coins applies faster region-based CNN, geometric constraints, and Residual Network (ResNet). A portable system allows visually impaired people to *detect and recognize Euro banknotes* [23]. The banknote detection is based on the modified Viola and Jones algorithms, while the banknote value recognition relies on the Speed Up Robust Features (SURF) technique. The Haar features and AdaBoost algorithms, proposed by Viola and Jones, are the core of the algorithm for the Euro banknote detection, while the Fast–Hessian matrix is the core of the SURF descriptor that is used for the detection of points of interest between images as well as to match them [23]. The accuracies of banknote detection and banknote value recognition are 84% and 97.5%, respectively.

A *deep learning object detector* [24] recognizes static and moving objects in real time. After detection, a Kalman Filter multi-object tracker is used to track these objects over time to determine the motion model. Motion features are extracted to quantify the level of hazard of a given object using five predefined classes with a neural network-based classifier.

#### 2.1.3. Face Recognition Systems

A real-time *face recognition system* for people with visual impairments is developed [25]. A camera sends a video to be processed; Haar-Like Features (HLF) Cascade is used as a classifier with AdaBoost to detect a face within an image; 42 characteristic points of the face are extracted using the Active Appearance Model (AAM), and a new hybrid model named Amαβ-CMKNN (Alpha-Beta Associative memories (Amαβ) with Correlation Matrix (CM) and K-Nearest Neighbors (KNN)) is used as a classifier to determine to whom a particular face belongs. 

Another *face recognition system*, based on computer vision algorithms (region proposal networks, ATLAS tracking and global, and low-level image descriptors) and deep convolutional neural networks [26], is able to detect, track, and recognize people from video streams in real-time. It includes a novel video-based face recognition framework that is able to construct an effective global, fixed-size face representation method with independence of the length of the image sequence.

*Mouth-based emotion recognition using deep learning* [27] helps people with disabilities who have difficulties seeing or recognizing facial emotions. The position and curve of the lips has been analyzed through CNNs. Four convolutional neural networks have been tested: VGG16, InceptionResNetV2, InceptionV3, and Xception. InceptionResNetV2 delivers the best result with an accuracy of 79.5%.

### 2.2. Hearing Impaired

The component designed for the hearing impaired is *sound recognition systems*. 

These components are introduced next.

#### Sound Recognition Systems

UbiEar [28] is a *smartphone-based sound awareness system* for the hearing impaired. The deaf person subscribes to one or multiple acoustic events (e.g., doorbell ring), described with a short introduction and a picture on the smartphone interface. A light-weight deep convolutional neural network enables location-independent acoustic event recognition. Event reminder includes, as options, a graphic view with the name of the acoustic event, followed by screen flickering, phone vibration, or flashing of the phone flashlight. 

The Deaf Assistance Digital System (DADS) [29] performs speech and *sound recognition* as well; the speech engine can recognize critical cautionary words, e.g., to prevent car or fire accidents using the Soundex algorithm; the sound engine can recognize four sound categories (vehicle, baby, normal, and loud sounds) using machine learning. Three different sound engine classifiers were tested: K-Nearest Neighbor (KNN), Neural Network (NN), and Decision Tree (DT); a three-layer NN with 60 nodes achieved the best results. Furthermore, a smart hearing aid *detects and makes audible important noises (e.g., fire alarm, car horn)* [30]. The designed deep neural network can treat input noise differently according to its classification (important, or not important). 

### 2.3. Physically Impaired

The components designed for the physically impaired are: (1) *human-machine interfaces* and (2) *human activity recognition systems*. These components are introduced next.

#### 2.3.1. Human-Machine Interfaces

A *human-machine interface* is proposed, which allows people with spinal cord injuries to use a computer [31]. The designed human-machine interface uses head movements and eye blinking for mouse control. The head mouse control is based on facial recognition. The head direction, as well as eye states (open, closed), are detected from the computer’s camera; a convolutional neural network transforms the head movements to the actual coordinates of the mouse; the mouse pointer is controlled with head movements and the mouse buttons with eye blinks.

*A Brain Computer Interface (BCI)* [32] integrates eye movement information into electroencephalographic (EEG) signals. EEG recordings are classified into six categories using a random forest algorithm. This way, it is possible to *move an electromechanical wheelchair* left, right, forward, and backward. 

*A hybrid brain-computer interface system for home automation control* is proposed [33]. It utilizes steady state visually evoked potentials (SSVEP) and eye blinks to provide 38 commands (i.e., 6 × 6 SSVEP commands and two eye blink commands) for controlling daily activities through a single bipolar channel. SSVEP signals provide selection functions, and single eye blinks provide the functionality to confirm selections. Resetting a selection requires one to perform a simple double eye blink. The short-time Fourier transform and convolution neural network algorithms are utilized for feature extraction and classification, respectively. The proposed system provides 38 control commands with a 2 s time window and a good accuracy (i.e., 96.92%) using one bipolar electroencephalogram (EEG) channel. 

An *online brain-computer interface video game* (MindGomoku) for entertaining people with disabilities is introduced [34]. It uses a simplified Bayesian convolutional neural network (SBCNN) for P300 signal classification and character recognition with good accuracy, even on a small training data set.

#### 2.3.2. Human Activity Recognition Systems

A *RFID-enabled data sensing tool* that uses machine learning *for human activity recognition* in ambient assisted living is described [35]. Human activity recognition is essential to assist a person with disability by understanding the context and adapting to the circumstances (emergency-related healthcare, wellbeing services, etc.). Passive RFID tags can recognize sequential and concurrent activities; a multivariate Gaussian algorithm for machine learning, that uses maximum likelihood estimation to classify and predict the sampled activities, is developed. 

A *deep learning model* named ST-DeepHAR [36] has also been designed for *human activity recognition* in the Internet of Healthcare Things. It uses a supervised dual-channel model that comprises long short-term memory (LSTM), followed by an attention mechanism for fine-tuned temporal feature selection with a convolutional residual network for the spatial fusion of sensor data. Furthermore, a *deep architecture based on the combination of convolutional and LSTM recurrent layers* performs activity recognition from wearable sensors [37]. The proposed model, which can perform sensor fusion naturally, does not require expert knowledge in designing features. It explicitly models the temporal dynamics of feature activations. It outperforms previous results in the OPPORTUNITY dataset of everyday activities by 4% on average and by 9% in an 18-class gesture recognition task. A *lightweight model* of a *deep neural network with CNN-LSTM for human activity recognition* is also proposed [38]. It extracts the features in an automated way and categorizes them according to some model attributes. It is highly robust and, with an accuracy of 97.89%, it provides better activity detection capability than traditional algorithms.

## 3. 5G for People with Disabilities

The fifth-generation wireless technology for digital cellular networks is known as 5G. It is expected to create smart networked communication environments where things, data, people, applications, transport systems, and cities are connected. 

**Figure 3:**
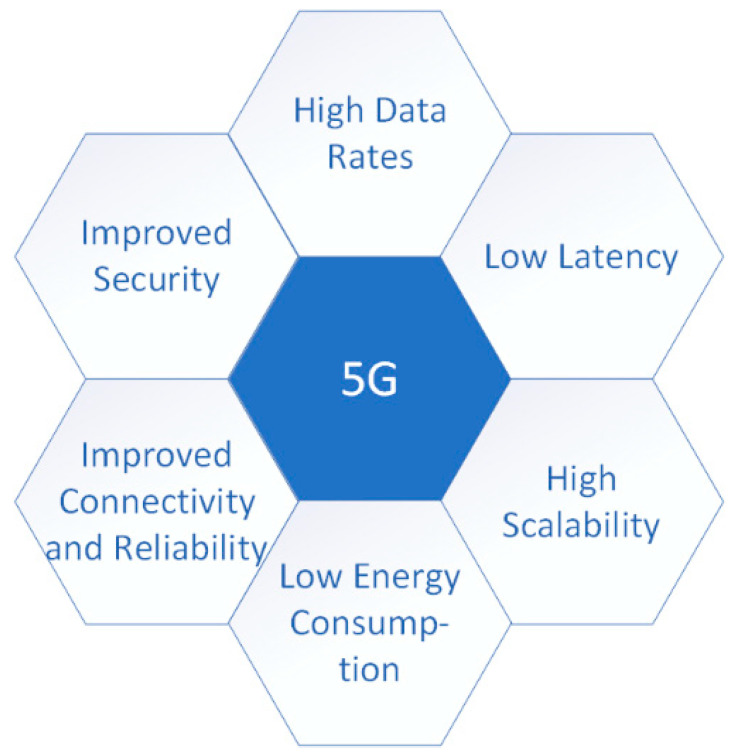
Key 5G capabilities for assistive technology.

Machine learning is particularly well-suited to operate with 5G networks [39,40] since it requires massive amounts of data to predict activity accurately, and 5G can transmit higher volumes of data faster than current networks. Therefore, future 5G characteristics could generate significant improvements in many scenarios that support machine learning and Internet of Things solutions for people with disabilities [41]. Figure 3 illustrates the key 5G capabilities that will have significant importance in improving assistive technology for people with disabilities. We discuss these key 5G capabilities next.

### 3.1. Low Latency

Low latency is an important requirement for computer vision applications. Computer vision systems need time to capture images, process them, and generate an alert signal. Additionally, 5G networks can reduce roundtrip latency to 1 ms [42]. Visually impaired applications with strict latency requirements, such as navigation systems [43], can benefit significantly. The same applies to sound recognition systems for the hearing impaired, designed to prevent accidents or alert about emergencies (e.g., fire alarm, car horn).

### 3.2. High Scalability

Network scalability is essential in the design of the next generation wireless communication networks. Similarly, 5G networks must be highly scalable to support many devices within an area. The ability to support a massive deployment of sensors/RFID-based devices in a scalable manner is also important for human activity recognition systems for the physically impaired.

### 3.3. Low Energy Consumption

Operational life time refers to the operation time per stored energy capacity. This is particularly important for navigation systems [44]; when their battery life is low, the autonomy of the visually impaired is seriously limited. The target is to implement low-cost devices with an extended battery. Battery charge or replacement should be delayed as much as possible.

### 3.4. Improved Connectivity and Reliability

Instantaneous connectivity, as well as robust and reliable connectivity solutions, is required in 5G to offer the best experience to highly mobile users. High reliability, as well as connectivity, are critical and should always be guaranteed for the successful development of navigation systems for the visually impaired and sound recognition systems for the hearing impaired.

### 3.5. Improved Security

Network security consists of the policies and practices adopted to prevent and monitor unauthorized access, misuse, modification, or denial of a computer network and network-accessible resources. Security is challenging in 5G due to new network services, heterogeneity of connected devices, requirements to support IoT and mission-critical applications, new stakeholders, increased number of users, and high user privacy concerns [45]. Network privacy refers to a variety of factors, techniques, and technologies used to protect sensitive and private data, communications, and preferences. It is required to provide robust and secure solutions for 5G to protect sensitive personal data from people with disabilities, since they need stronger security guarantees.

### 3.6. High Data Rates

The current 4th Generation Long Term Evolution (4G LTE) system supports up to 100 Mbit/s download speeds. Future 5G networks will be able to support higher frequencies (including those higher than 10 GHz). Therefore, transmission with higher rates (on the order of Gbps) will be achieved. Additionally, 5G needs to support applications with high data rates to provide a satisfactory experience to the users, such as real-time video/telepresence for remote healthcare to enable individualized consultations, treatment, and patient monitoring for people with disabilities from their smart homes.

On the other hand, the International Telecommunication Union (ITU) has identified three different usage scenarios/use cases for International Mobile Telecommunications-2020 (IMT-2020) systems: *enhanced Mobile Broadband (eMBB)*, *massive Machine Type Communications (mMTC)*, and *Ultra Reliable and Low Latency Communications (URLLC)* [46]. They can be summarized as follows:

- *Enhanced Mobile Broadband (eMBB)*: it addresses use cases for access to multi-media content, services, and data. This usage scenario covers a range of cases, including wide-area coverage and hotspots.
- *Massive Machine Type Communications (mMTC)*: this use case is characterized by a very large number of connected devices, typically transmitting a relatively low volume of non-delay sensitive data. Devices are required to be low cost and have a very long battery life.
- *Ultra Reliable and Low-Latency Communications (URLLC)*: this use case has stringent requirements for capabilities such as latency, reliability, and availability. Some use cases include wireless control of industrial manufacturing or production processes, remote medical surgery, and transportation safety.

## 4. Application Scenarios and Benefits of Machine Learning and 5G for People with Disabilities

**Figure 4:**
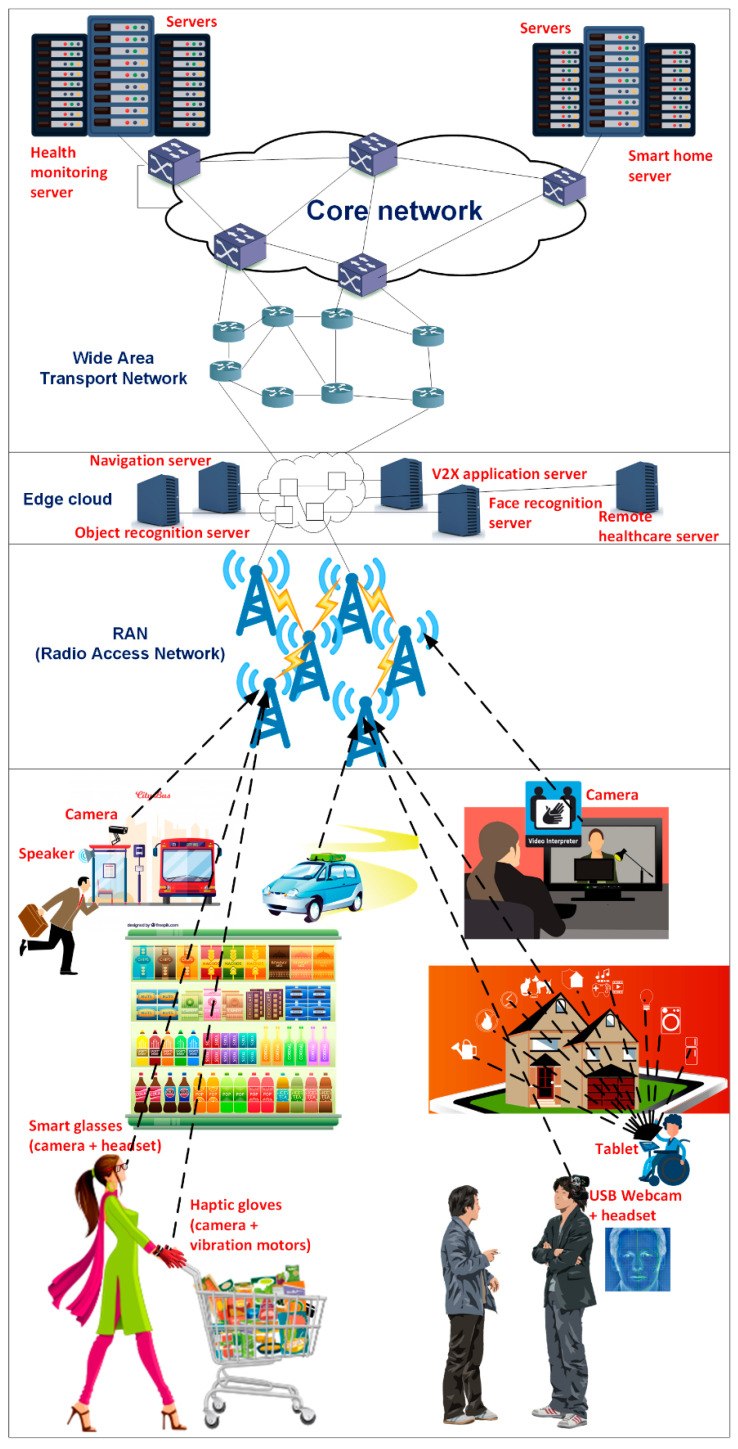
Different application scenarios for the integration of machine learning and 5G for people with disabilities.

Next, several application scenarios and the main benefits of machine learning and 5G for people with disabilities are discussed (see Figure 4).

### 4.1. Shopping

Fortunately, 5G offers greater network speeds and capacity to deal with the biggest challenge, being able to keep up with the real-time demands of the user [47]. Smart glasses (with a camera and a headset) and haptic gloves (with vibration motors and cameras, as well) analyze images to accurately locate and identify objects; they use computer vision technology to transfer real-time visual information into auditory information to assist the visually impaired do their daily activities such as shopping [47,48], which increases their autonomy and self-confidence. This way, a pair of glasses helps the visually impaired to buy groceries in a market. It is possible to capture images from the products using a camera, classify them using convolutional neural networks, and return the prices. 

### 4.2. Travelling

With 5G and machine learning, bus and train routes will be easily accessed so that the visually impaired can move freely; with facial recognition systems [24], they can maintain social contacts easily. Additionally, 5G connected self-driving cars [49] are a practical transportation option for those people with disabilities who cannot drive. Remote driving [50,51] enables a remote driver or a Vehicle-to-everything (V2X) application to operate a remote vehicle for those passengers who cannot drive themselves; high-definition video cameras, installed in the vehicle, send multiple real-time video feeds towards a V2X application server, providing a high-degree view of the vehicle’s surroundings over a high-bandwidth 5G network; this mode supports vehicular speeds up to 250 km/h.

### 4.3. At Work

American Sign Language (ASL) is a natural predominant sign language of deaf and hard hearing communities in the United States and most of anglophone Canada. It is expressed by movements of the hands and face. ASL can be recognized using deep learning and computer vision [52] to allow natural communication between sign and non-sign language speakers. With 5G networks up to 100 times faster than 4G, the latency problem is eliminated so that all users benefit from videoconferencing for a better communications experience; consequently, employment opportunities for the hearing impaired are increased. 

Smart assistance systems help the visually impaired by converting visual data using video and image processing. A lightweight Convolutional Neural Network (CNN)-based 2.5D object-recognition module [53] provides a depth image-based object-detection method to classify obstacles and give location and orientation information; furthermore, optical character recognition (OCR) is used to detect and read text from images captured by the camera.

### 4.4. Smart Homes

5G real-time services allow people with disabilities to live independently in connected smart homes since 5G networks transmit a significant amount of data at very high speeds. Neural networks can improve home automation, comfort, security [54], and health care for people with disabilities in their smart homes [55]. Neural networks can implement virtual sensors to replace temporarily unavailable physical sensors for indoor localization systems [56]. These systems are helpful for monitoring and assisting people with disabilities during critical situations, such as wandering or falling. Neural networks [57] can learn the users’ habits and activities; this way, their next moves are predicted, and alerts are sent (e.g., time to take a given medicine, fire alarm). The faster speed and lower latency of 5G improves security camera systems significantly and provides enough capacity to connect faster and more efficient Internet of Things (IoT) devices. 

## 5. 5G Service-Based Architecture

**Figure 5:**
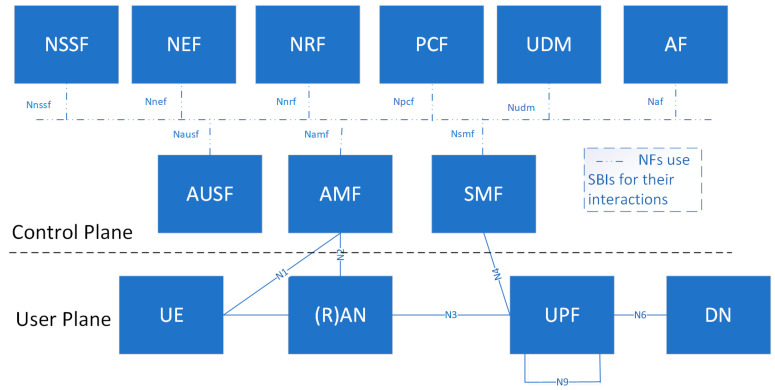
Service-based representation of the 5G non-roaming system architecture.

Next, the service-based representation of the 5G non-roaming system architecture, specified by 3GPP [58], is depicted in Figure 5. We distinguish between the User Plane (UP) and the Control Plane (CP). The UP carries only user traffic while the CP is dedicated to network signaling. The 5G system architecture aims at separating the UP and the CP functions to allow scalability and flexible deployments.

In the user plane, we observe that the User Equipment (UE) is connected to either the Radio Access Network (RAN) or a non-3GPP Access Network (e.g., Wireless Local Area Network, WLAN) and to the Access and Mobility Management Function (AMF) as well. RAN refers to a base station with new radio access technologies and evolved LTE. 

The upper part of the figure is the 5G core network CP and has a ‘bus’; network functions (NFs) (e.g., AMF) within the CP allow any other authorized NFs to access their services using service-based interfaces (SBIs) for their interactions. 

The 5G system architecture consists of the following NFs, which constitute the 5G core network:

- *Access and Mobility Management Function (AMF)*: it is responsible for registration and connection management as well as mobility management; it also supports authentication and authorization.
- *Session Management Function (SMF)*: it supports session management as well as User Equipment (UE) IP address allocation and management; it also selects and controls User Plane Functions (UPFs) for data transfer.
- *User Plane Function (UPF)*: it is responsible for packet routing and forwarding; acts as an external PDU session point of interconnect to Data Network (DN), e.g., to operator services, Internet access or third party services; it is also the anchor point for intra- and inter- Radio Access Technology (RAT) mobility.
- *Policy Control Function (PCF)*: it provides policy rules to control plane functions (e.g., AMF, SMF)
- *Application Function (AF)*: it interacts with the core network to provide several services.
- *User Data Management (UDM)*: it stores and manages subscription data of the UE; it supports access authorization based on subscription data.
- *Authentication Server Function (AUSF)*: it acts as an authentication server and stores data for the authentication of UEs.
- *Network Slice Selection Function (NSSF)*: it selects the set of network slice instances serving the UE.
- *Network Exposure Function (NEF)*: it supports exposure of the services and capabilities provided by 3GPP NFs and a secure provision of information from external application to 3GPP network; it translates internal/external information.
- *NF Repository Function (NFR)*: it supports service discovery function, maintains NF profile, available NF instances, and their supported services.

Furthermore, the NF modularization design enables flexible and efficient network slicing.

This representation also includes point-to-point reference points (e.g., N4) for the interaction between pairs of NFs when necessary.

## 6. A 5G Network Slicing Architecture for People with Disabilities

Additionally, 5G can offer a wide range of network services with different performance requirements. A network slicing architecture allows network operators to support heterogeneous services. Network slicing consists of dividing the physical network into multiple logical networks (network slices); each logical network is optimized to provide the required network capabilities for a specific application/service. Network slicing opens new business opportunities for mobile operators, since they are able to split their physical network resources into multiple logical slices and lease these slices out to interested parties.

Network slicing divides the physical network into multiple virtualized logically isolated end-to-end networks. A ‘5G slice’ is composed of a collection of 5G NFs and specific RAT settings that are combined for a specific use case. A 5G slice can span all domains of the network: access network, transport network, core network, and terminal domains [59]. Network slicing services can be automatically generated, maintained, or terminated according to services’ requirements. Therefore, network slicing is a flexible and scalable solution when the same operator network needs to accommodate different use cases, each with its own service requirements. For this reason, it has attracted the attention of academia and industry alike.

**Figure 6:**
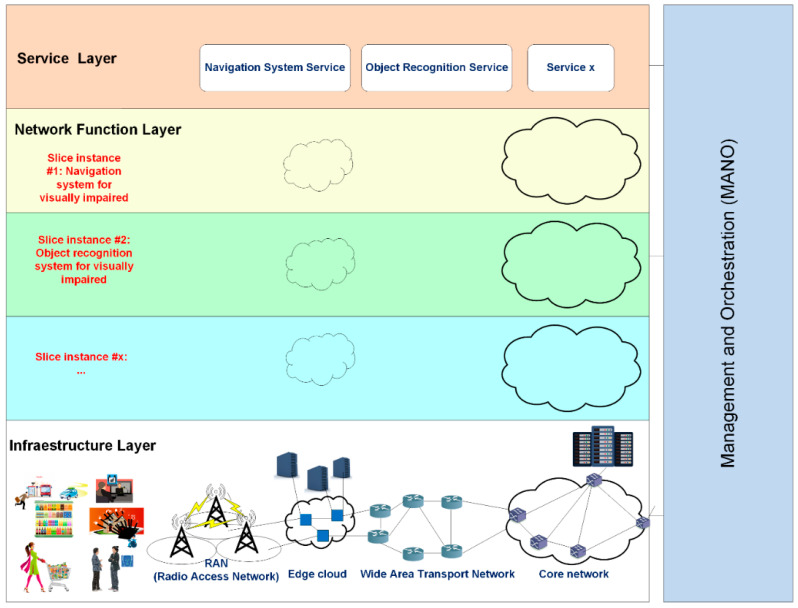
Network slicing architecture for people with disabilities.

Network operators can also deploy, flexibly and quickly a wide range of services for applications that target people with disabilities using network slicing. Next, we introduce the main design elements of our devised 5G network slicing architecture for people with disabilities (see Figure 6).

***Infrastructure layer***: it refers to the physical network infrastructure, which includes the RAN, the edge cloud, the wide area transport network, and the core network. It provides the control and management of the infrastructure, and allocates resources (computing, storage, bandwidth, etc.) to network slices so that the higher layers can access and handle them appropriately. Since slices may span multiple domains, this layer comprises the infrastructure of multiple operators [60]. 

***Network function layer***: it executes all the operations to manage the virtual resources and the life cycle of the network functions. It enables the optimal assignment of the virtual resources to the network slices, and chains multiple slices together to provide a specific service or application [61]. Two fundamental technological enablers of this layer are virtualization of network functions (Network Function Virtualization, NFV) [62], as well as software-defined, programmable network functions (Software Defined Networking, SDN) [63]. NFV is a technology used to virtualize complex hardware-based network node functions into software building blocks that can be connected and chained to create advanced communication services; these flexible software blocks are called VNFs (Virtualized Network Functions). SDN (Software-Defined Networking) technology is an approach to network management that decouples the network control, data forwarding paths, and functions. This way, the SDN architecture enables the network control to become directly programmable and the underlying infrastructure is abstracted from applications and network services.

***Service layer***: The service layer represents the services (end-user or business services) to be supported. Each service is represented by a service instance and is provided by a network operator or a third party. A Network Slice Instance (NSI) is a managed entity created by an operator’s network with a lifecycle independent of the lifecycle of the service instance(s) [64]. An NSI provides the network characteristics (e.g., ultra-low-latency, ultra-reliability) required by a service instance. An NSI may also be shared across multiple service instances provided by the network operator. The NSI lifecycle typically includes an instantiation, configuration and activation phase, a run-time phase and a decommissioning phase. During the NSI lifecycle the operator manages the NSI. Each service should satisfy the SLA (Service Level Agreement) requirements such as throughput or latency.

***Management and Orchestration (MANO)***: It implements three key functional blocks:

NFV Orchestrator (NFVO), VNF Manager (VNFM), and Virtualized Infrastructure Manager (VIM). VIM manages the lifecycle of virtual resources, as requested by the NFVO in an NFV Infrastructure (NFVI) domain. VNFM manages the lifecycle of VNFs. NFVO manages the resources from different VIMs and the creation of an end-to-end service that involves VNFs from different VNFM domains.

### 6.1. People with Disabilities Slices Design

Network slicing in 5G networks enables that, in the ecosystem of people with disabilities, different tenants (customers) issue requests to an infrastructure provider for acquiring network slices to cope with a wide range of use cases. 

The most relevant Key Performance Indicators (KPIs) of the different use cases, for people with disabilities, are summarized in Table 1.

The end-to-end latency has been measured as the time it takes to transfer a given piece of information from a source to a destination, measured at the application level, from the moment it is transmitted by the source to the moment it is received at the destination.

Additionally, 3GPP has classified 5G use cases into three categories: eMBB, mMTC, and URLLC (see Section 3.6). Although it is not clear cut to which category a use case belongs to [68], based on the main KPIs and functional requirements of the different use cases for disabled, we propose the following slices:

- *Navigations systems for the visually impaired*: URLLC requirements.
- *Object recognition systems for the visually impaired*: URLLC requirements.
- *Facial recognition systems for the visually impaired*: URLLC requirements.
- *Sound recognition systems for the hearing impaired*: URLLC requirements.
- *Videoconference with ASL recognition*: eMBB requirements.
- *Smart homes for handicapped (see Section 4.4)*: We distinguish between different areas that require automation and control in smart homes [55]:
  ◦ *Health monitoring*: falls and balance, first aid and emergence service, vital signs monitoring, telemedicine, etc.
  ◦ *Behavior monitoring (to learn the user’s habits and alert caregivers and family members of any significant changes*): medication habits, diet, sleeping patterns, etc.
  ◦ *Security*: intruder detection, fire alarm, automatic door lock, kitchen equipment control, etc.
  ◦ *Environment (home automation and smart appliances)*: AC control, heating control, water temperature control, light control, etc.
  ◦ *Entertainment*: TV on/off, channel/movie selection, daily exercise, etc.
  The proposed smart home 5G network consists of *three slices*:
  ◦ *A healthcare and behavior monitoring network slice [69]*: it is reserved for the use of healthcare professionals and caregivers to help the disabled person (remote healthcare); it has eMBB requirements.
  ◦ *A security slice*: to ensure isolation, this slice has its own VNFs for the access and core network; it also has its own AUSF for the authentication of devices before they gain access to the slice; it has URLLC requirements.
  ◦ *An environment and entertainment slice*: it provides network connectivity to the sensors, actuators (home appliances), and wearables for the disabled; it has mMTC requirements.
- *Remote driving for people with disabilities (see Section 4.2)*: self-driving cars don’t require human involvement. Road information and controlling messages will be exchanged mainly between adjacent vehicles (Vehicle-to-vehicle (V2V) communications) with very low latency (e.g., 1 ms) [70]; network coverage (Vehicle-to-network, V2N) is also required, with nearly 100% reliability, to avoid traffic accidents. Video exchange between vehicles (V2V), and between vehicles and the infrastructure (Vehicle-to-infrastructure, V2I), will also be required for safety purposes in certain scenarios where high data rates are required [70].

Furthermore, remote driving [50] (see Section 4.2) enables a remote driver or a V2X application to operate a remote vehicle for those passengers who cannot drive themselves; low-latency and high reliability need to be supported with the aid of a V2X application server located at the network edge. The associated remote driving slice has URLLC requirements.

### 6.2. Benefits of a Network Slicing Architecture for People with Disabilities 

Next, we introduce the main benefits of the network slicing architecture for people with disabilities:

Network slicing improves the Quality of Service (QoS) of the different applications for people with disabilities allocating dedicated slices for each use case. The 5G use cases can be clustered into three major categories (as discussed in Section 3.6): eMBB, mMTC, and URLLC. In the ecosystem of people with disabilities, the different applications have been classified as one of the three different types of slices depending on their specific requirements (see Section 6.1): low latency, low loss rate, high reliability, etc. For instance, mMTC applications such as sensors, actuators (home appliances), and wearables for the disabled at home (see Section 6.1) typically have a multitude of low throughput devices, whereas navigation systems for the visually impaired or remote driving in the URLLC category require extremely low latency and very high reliability. In addition, remote healthcare for the disabled in eMBB category requires very high bandwidth to transmit a large amount of content. 

The traditional mobile communication networks that provide services based on the “one-size-fits-all” design are usually built on specialized equipment and dedicated platforms with weak flexibility and scalability. Therefore, they cannot address the wider range of performance and cost requirements of the new use cases for people with disabilities. On the other hand, 5G network slices, which are represented by logically isolated and self-contained networks, are flexible enough and highly customizable to accommodate diverse use cases, simultaneously, over the same network infrastructure [71]. It is possible to provide network services efficiently when the large monolithic network functions of the traditional networks, based on specific hardware, are decomposed into several software, based smaller modular network functionalities with varying granularity [71]. These 5G network functions can be combined appropriately with a suitable 5G RAT on demand to form different network slices that are tailored to specific use cases. 

Existing QoS architectures like Differentiated Services (DiffServ) [72] can discriminate different types of traffic (e.g., VoIP traffic, high-definition video, web browsing, etc.), but they can’t discriminate and treat the same traffic (e.g., VoIP traffic) coming from different tenants in a different way. In contrast, network slicing provides an end-to-end virtual network for a given tenant. 

Furthermore, network slicing can improve the scalability of the network as service requirements and the number of users change. The amount of traffic that flows through the network is not always the same. IoT devices for people with disabilities (wearables, cameras, etc.) operate in sleep and wake cycles, which causes network resources to be idle or over-utilized sometimes. Network slicing enables the dynamic allocation of idle network resources of a particular network slice to another slice that demands high resource requirements. Dynamic resource allocation enables the accomplishment of the QoS requirements even during congestion situations. This technique improves the efficiency of resource utilization and enhances the network performance. Finally, network slicing improves reliability, security and privacy, since it isolates the network resources of one service from the others. Reliability is improved because faults or errors in one slice don’t affect the proper functioning of the rest. Slice isolation ensures that the security attack on one slice doesn’t affect the others. Allocating separate slices for different applications with strong slice isolation mechanisms allows better protection of sensitive information from the disabled.

## 7. Research Challenges

Next, the research challenges for machine learning and 5G for people with disabilities are introduced.

*Customization for people with disabilities is a key challenge*. Since people with disabilities have special needs, machine learning assistive technologies should be adapted to their different vision of the world. The development of applications for people with disabilities is usually hindered by the scarcity of training data for a specific population (e.g., there are very few sign language videos for the deaf). A novel approach is that users train their own assistive devices with their own data; this way, they can personalize their own data-driven assistive technology and improve its accuracy. For example, a mobile sound detector is trained by people with hearing impairments with their own personally relevant sounds (filtering out unimportant ones), or the visually impaired can train an object recognizer with their own objects. New innovative ways should be designed to provide feedback about the quality of their collected training data.

*Collecting big data sets is also challenging. Training algorithms used by applications for people with disabilities is time and effort-intensive* because it involves acquiring images/audio, and other types of data, under different conditions. For instance, the number of objects that a system developer uses to train an object recognition system is going to be a small subset compared to the thousands of objects that the system will be able to recognize. Although blind people have the option to train the system with their own objects, they may abandon it due to the large amount of time and effort it takes. This issue can be solved by sharing resources through crowdsourcing, although a special effort must be made to combine datasets from different sources. A possible solution to this problem would be the maintenance of a database of training data by systems developers that would incorporate additional data to train objects sent by current users.

*Another challenge is how to optimize the recognition accuracy of the machine learning algorithms in real-time applications for the disabled.* There is a tradeoff between the obtained classification accuracy and the maximum delay that the application can tolerate to maintain its quality of service. For example, the recognition accuracy for moving a wheelchair based on EEG eye movement recognition may be improved computing more spectral and statistical features, but the addition of more features from 32 channels would require a feature selection method and would increase the computational burden so much that the proposed method would not work as a real-time application [32]. Sometimes latency is increased due to the congestion and the distance of the network systems. In these cases, instead of offloading computation to the remote cloud, edge computing can be leveraged in 5G networks to store and process content near mobile users [73]. The decentralization of cloud computing infrastructure to the edge introduces new challenges to 5G evolution [73], such as resource management to support only a constrained number of applications with moderate needs because of the limited computing and storage resources in an individual Mobile Edge Computing (MEC) platform or fair resource sharing and load balancing.

*Slice security* is also required. Dynamic resource sharing among tenants enables more efficient network resource usage but also introduces new problems such as how to guarantee a secure isolation of the slices. Security requirements of specific tenant applications, such as remote vehicular control (for people with disabilities), could restrict how the slices are partitioned, or even prevent the coexistence of network slices that would share the same hardware [56]. Since network slices exchange important information about the network state and the network resources, the higher the number of virtual network functions share between slices, the higher the security risks might be [74].

Finally, another important challenge is *how to implement network slicing in 5G networks to satisfy the diversified service requirements.* The complexity and difficulty of dynamic slice creation and management increases with the number of services and applications. The life-cycle management of the network slices is a critical issue that demands that network operators deploy virtual network functions and allocate network resources to build network slices rapidly and dynamically depending on the service load. When virtual networks are created, traditionally a set of the expected amount of network resources (e.g., networking, processing/computing, and storage) is allocated; these network resources tend to be either over-provisioned or under-provisioned. This usually happens when the number of users of the network slices is not known, and there are insufficient resources as the number of users increases, thereby resulting in poor network performance. Therefore, efficient network slice resource allocation algorithms have to incorporate a form of Pareto optimality techniques, whereby network slice resources can be dynamically scaled up/down or in/out to optimally serve the total number of slice consumers without leaving any other active network slice with insufficient amount of resources, i.e., negatively impacting the performance of any active network slices [74].

## 8. Conclusions

In this paper, an overview of machine learning and 5G, for people with disabilities, is provided. Machine learning offers new ways to help people with disabilities. The most important machine learning accessibility tools for people with disabilities have been introduced. The components designed for the visually impaired are: (1) *navigation systems*, (2) *object recognition systems*, and (3) *face recognition systems*. The one designed for the hearing impaired is *sound recognition systems*. Finally, the components designed for the physically impaired are: (1) *human-machine interfaces* and (2) *human activity recognition systems*.

Machine learning is particularly well-suited to operate with 5G networks since it requires massive amounts of data to predict activity accurately and 5G can transmit higher volumes of data faster. The key 5G capabilities that will have significant importance in improving assistive technology for the disabled are low latency, high scalability, low energy consumption, improved connectivity and reliability, improved security, and high data rates.

The relevant application scenarios and main benefits have been described. Machine learning and 5G help people with disabilities remain autonomous in four basic scenarios: shopping, travelling, work, and smart homes. 

A 5G network slicing architecture has been proposed, and its main benefits have been discussed. Specific slices have been proposed based on the main KPIs and functional requirements of the different use cases for people with disabilities. The research challenges have also been surveyed, with particular reference to the improvement of training data, for people with disabilities. In this area, significant efforts are required. The traditional mobile communication networks that provide services based on the “one-size-fits-all” design are usually built on specialized equipment and dedicated platforms with weak flexibility and scalability. Therefore, they cannot address the wider range of performance and cost requirements of the new use cases for people with disabilities. For this reason, we propose a 5G network slicing architecture that improves the Quality of Service (QoS) of the different applications for people, with disabilities allocating dedicated slices for each use case. The 5G network slices, which are represented by logically isolated and self-contained networks, are flexible enough and highly customizable to accommodate diverse use cases simultaneously over the same network infrastructure. As future work we propose to conduct an empirical evaluation of the 5G architecture for people with disabilities to verify its effectiveness. 

## Figures and Tables

**Table 1:** KPIs of the different use cases for people with disabilities [50,65,66,67].

Use Case		End-to-End Latency	Data Rate (Uplink/Downlink)	Reliability
Navigations systems for visually impaired		10 ms	25 Mbit/s/1 Mbit/s	99.999%
Object recognition systems for visually impaired		<20 ms	25 Mbit/s/1 Mbit/s	99.999%
Facial recognition systems for visually impaired		10 ms	25 Mbit/s/1 Mbit/s	99.999%
Sound recognition systems for hearing impaired		<20 ms	1.5 Mbit/s/10 Mbit/s	99.999%
Videoconference with ASL recognition		4 ms	500 Mbit/s/1 Gbit/s	95%
Smart homes (UE to network)	Home appliances, electronic UE (e.g., printers) or IoT devices (e.g., smart flower to water a plant)	10 ms	500 Mbit/s/1 Gbit/s	95%
	Wearables (sensors embedded in clothing, UE monitoring biometrics)	10 ms	10 Mbit/s/10 Mbit/s	95%
	Real-time video/telepresence for remote healthcare	100 ms	40 Mbit/s/40 Mbit/s	99.999%
Remote driving (information exchange between a UE supporting V2X application and a V2X application server)		5 ms	25 Mbit/s/1 Mbit/s	99.999%

## Data Availability

Not applicable.
